# Supplementary material for: DPPIV+ fibro-adipogenic progenitors form the niche of adult skeletal muscle self-renewing resident macrophages
Source: Nat Commun. 2023 Dec 13;14:8273. doi: 10.1038/s41467-023-43579-3 (PMC10719395; doi:10.1038/s41467-023-43579-3)
Supplement: Supplementary file 1 — Supplementary file [file 41467_2023_43579_MOESM1_ESM.pdf]

DPPIV$^+$ fibro-adipogenic progenitors form the niche of adult skeletal muscle self-renewing resident macrophages

Farshad Babaeijandaghi, Nasim Kajabadi, Reece Long, Lin Wei Tung, Chun Wai Cheung, Morten Ritso, Chih-Kai Chang, Ryan Cheng, Tiffany Huang, Elena Groppa, Jean X. Jiang, Fabio M V Rossi

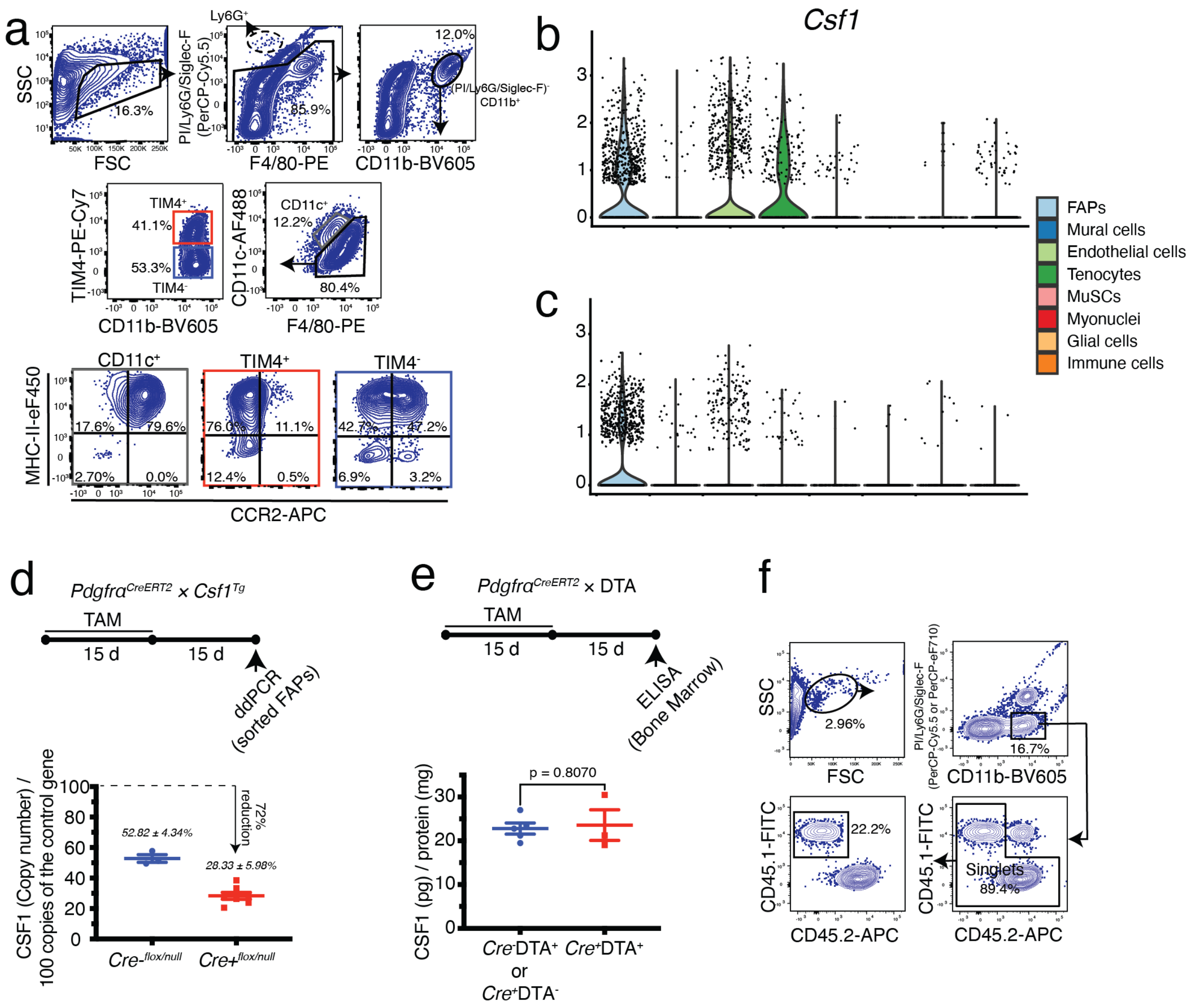

fig. S1

a) Flow cytometric analysis of resident myelomonocytic populations in skeletal muscle at steady state. Ly6G$^+$ neutrophils constitute a small population at the top-middle position. Siglec-F$^+$ eosinophils are present in very limited numbers in muscle at steady state, and they have already been excluded based on our initial FSC/SSC gating strategy, as they exhibit higher SSC values. **b)** Violin plots depicting expression of *Csf1* by different muscle resident cells from the Tabula Muris dataset and **c)** De Micheli AJ et al. **d)** Assessment of the recombination efficiency of the *Pdgfrα*^{CreERT2} × *Csf1*$^{flox/null}$ system using droplet digital PCR. The copy number of the *Csf1* gene was normalized to the *Ctnnb1*. FAPs sorted from TAM-induced Cre$^-$ × *Csf1*$^{flox/null}$ mice were used as a control to demonstrate the expected deletion of one allele of *Csf1* in the cells (each dot represents one mouse, data were pooled from 2 experiments). **e)** The amount of CSF1 detected by ELISA in the bone marrow of TAM-induced *Pdgfrα*^{CreERT2} × DTA mice compared to Tg control (each dot represents one mouse, data were pooled from $\geq$ 3 experiments, unpaired t-test). **f)** Representative plots of flow cytometric analysis of blood chimerism in *Csf1*^{flox} mice (recipient) surgically paired with B6 CD45.1 mice (donor).

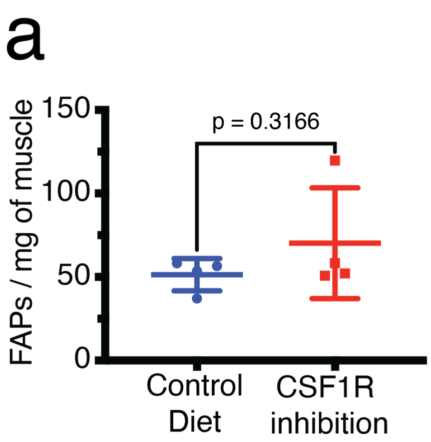

fig. S2

a) The number of FAPs detected by flow cytometry in TA muscle following CSF1R inhibition with PLX5562 (each dot represents one mouse, one experiment, unpaired t test).

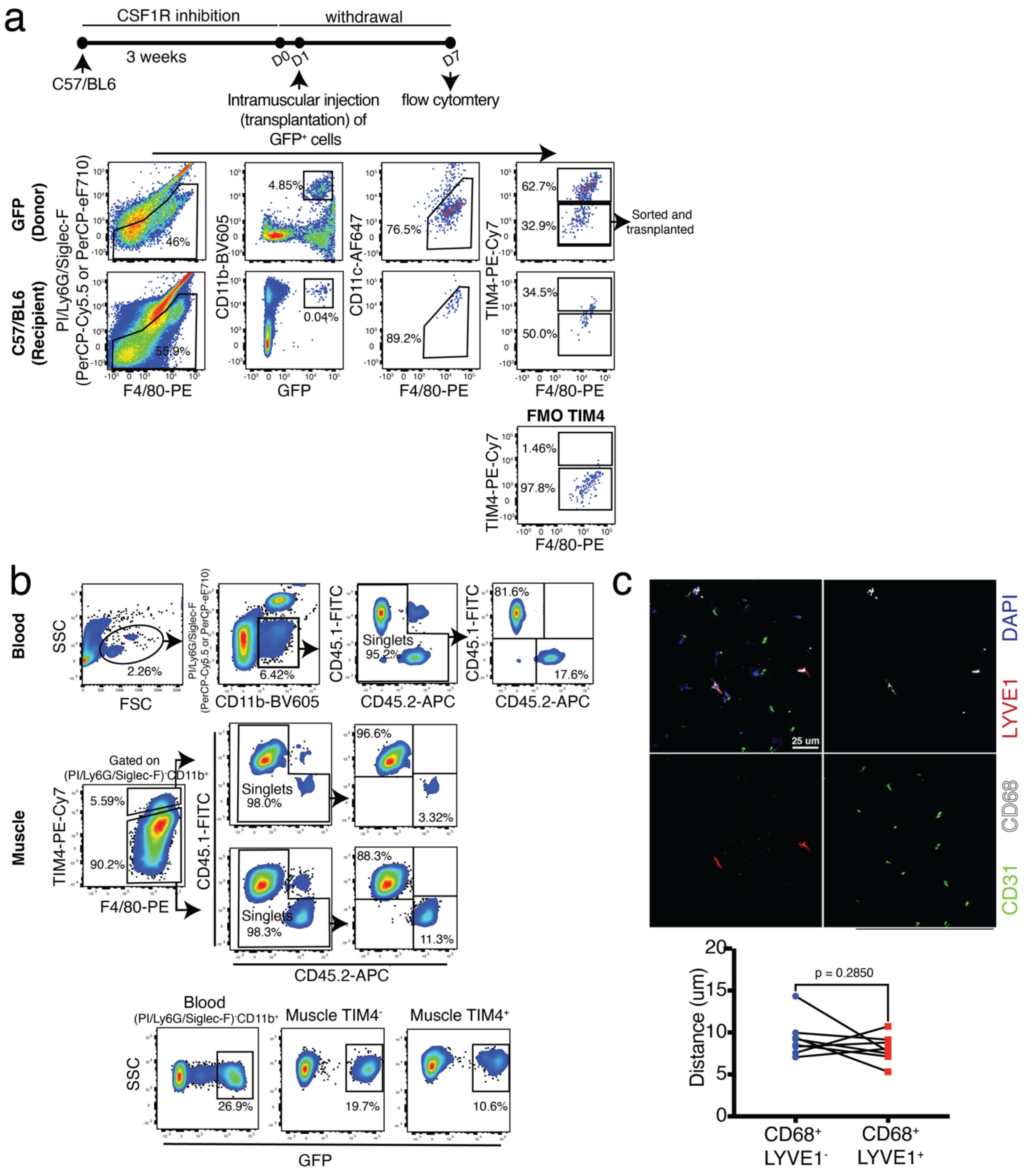

fig. S3

a) Experimental strategy to sort GFP$^+$TIM4$^-$ RMs for transplantation into a C57BL/6J mouse: A C57BL/6J mouse was treated with the CSF1R inhibitor to empty the niche of RMs for subsequent engraftment. One day after withdrawal of CSF1R inhibition, PI$^-$Ly6G$^-$Siglec-F$^-$CD11b$^+$GFP$^+$CD11c$^-$F4/80$^+$TIM4$^-$ cells, sorted from a GFP donor (top flow cytometry panel), were transplanted into the TA muscle. Six days later, the TA was collected to assess TIM4 expression by the transplanted GFP$^+$ cells using flow cytometry (bottom flow cytometry panel).

b) Representative plots of flow cytometric analysis of the extent of contribution from bloodborne monocytes to $TIM4^+$ cells following CSF1R inh/wd using parabiotic pairs of B6 CD45.1 mice (recipient) and C57BL/6J (CD45.2, donor, top panels) or C57BL/6J mice (recipient) and mice ubiquitously GFP (donor, bottom panel). **c)** Immunofluorescent staining of TA muscle sections and quantification of the distances between $LYVE1^+$ SRRMs or $LYVE1^-$ RMs and endothelial cells (n = 9 mice pooled from 2 experiments; paired t-test). On average, we manually measured the distances of approximately 30 $CD68^+LYVE1^-$ and 15 $CD68^+LYVE1^+$ RMs from their closest $CD31^+$ cells in each mouse.

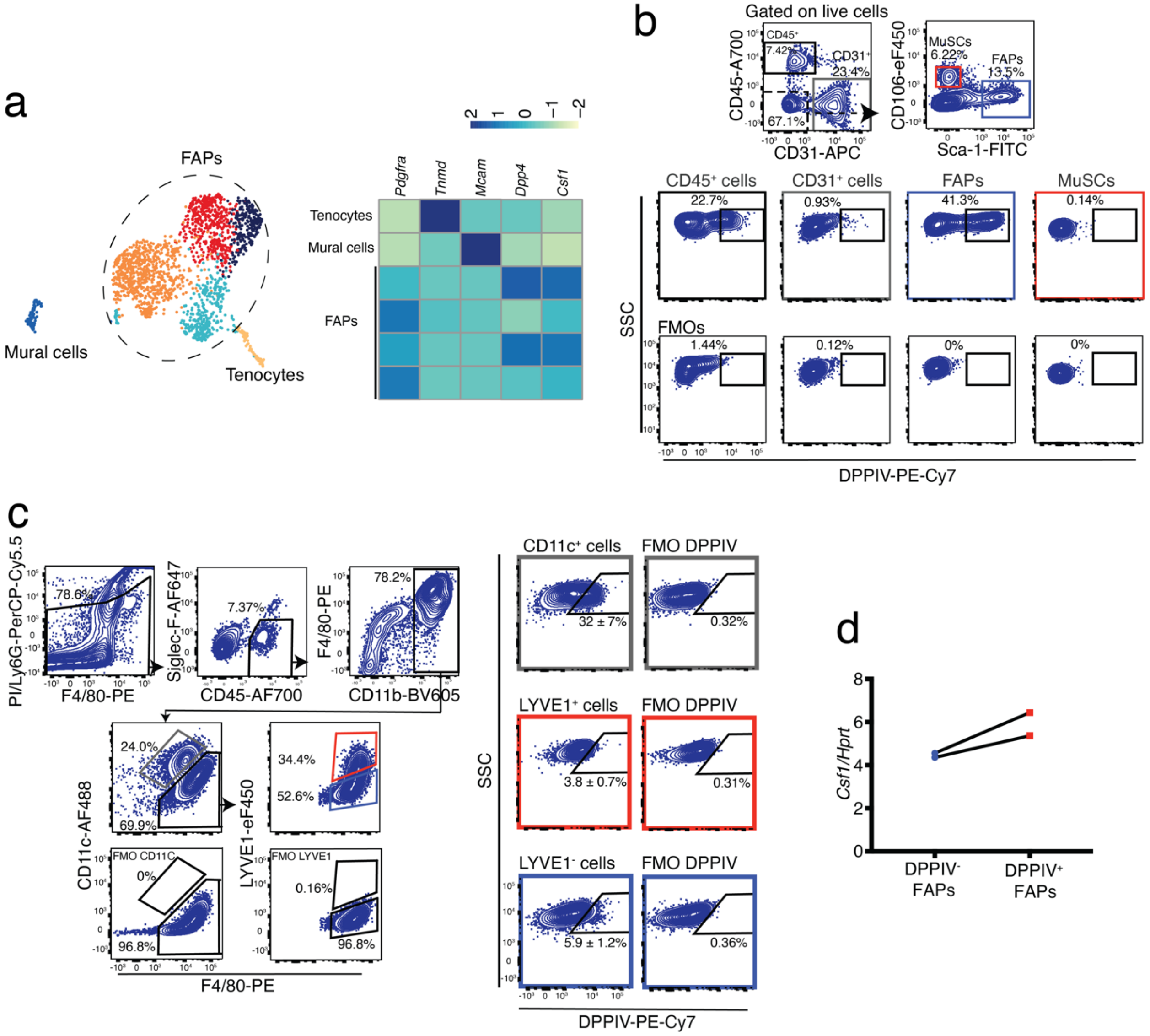

fig. S4.

a) Feature plot depicting different stromal populations in skeletal muscle (left) and heatmap showing expression of *Csf1* and key genes in FAPs compared to the other cell populations (right). **b)** Flow cytometric analysis of the expression of DPPIV on different muscle resident cells (n = 1 mouse) followed by **c)** Flow cytometric analysis of the expression of DPPIV on muscle resident myelomonocytic cells (n = 4 mice). **d)** *Csf1* expression by DPPIV$^-$ and DPPIV$^+$ FAPs at steady state assessed by droplet digital PCR (n = 2 mice, one experiment).

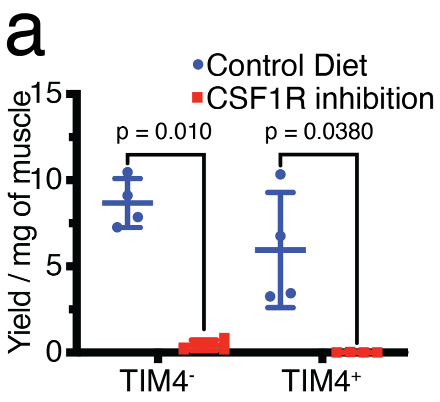

fig. S5.

a) The number of TIM4^{-} and TIM4^{+} RMs detected by flow cytometry in skeletal muscle following 3 weeks of CSF1R inhibition with PLX5562 (n = 4 mice per group, one experiment, unpaired t test with Welch's correction)

Antibody	Fluorophore	Clone	Dilution	Provider	Cat number
Anti-TIM4	PE-Cy7	RMT4-54	1:200	BioLegend	130010
Anti-LYVE1	eFluor™ 450 (eF450)	ALY7	1:500	Invitrogen	48-0443-82
Anti-CD45	Alexa Fluor® 700 (AF700)	30-F11	1:500	BioLegend	103128
Anti-CD11b	Brilliant Violet 605™ (BV605)	M1/70	1:500	BioLegend	101257
Anti-CD170 (Siglec-F)	PerCP-eFluor™ 710	1RNM44N	1:500	Invitrogen	46-1702-82
Anti-CD170 (Siglec-F)	Alexa Fluor® 647 (AF647)	E50-2440	1:400	BD Pharmingen™	562680
Anti-MHC Class II (I-A/I-E)	eFluor™ 450 (eF450)	M5/114.15.2	1:500	eBioscience™	48-5321-82
Anti-CD11c	Alexa Fluor 488 (AF488)	N418	1:500	AbLab	48-0051-01
Anti-CD11c	Alexa Fluor 647 (AF647)	N418	1:4000	AbLab	67-0051-01
Anti-CD192 (CCR2)	APC	SA203G11	1:200	BioLegend	150627
Anti-LY6G	PerCP-Cy™5.5	1A8	1:500	BD Pharmingen™	560602
Anti-F4/80	PE	BM8	1:200	Invitrogen	12-4801-82
Anti-CD106	Biotin	MK1.9	1:500	AbLab	31-0057-01
Anti-Sca-1	PE-Cy7	D7	1:4000	eBioscience™	25-5981-82
Anti-Sca-1	FITC	D7	1:500	eBioscience™	11-5981-82
Anti-CD31	APC	MEC 13.3	1:500	BD Pharmingen™	551262
Anti-DPP4	PE-Cy7	H194-112	1:200	BioLegend	137810
Anti-CD45.2	APC	104	1:400	eBioscience™	17-0454-82

Anti-CD45.1	FITC	A20	1:500	eBioscience™	11-0453-85
Streptavidin	Alexa Fluor® 488 (AF488)		1:200	BioLegend	405235
Streptavidin	eFluor™ 450 (eF450)		1:200	eBioscience™	48-4317-82
Anti-Fcγ Receptor		24G2	1:200	AbLab	21-0041-05
Anti- DPP4		Polyclonal	1:200	R&D Systems	AF954
Anti-CD31/PECAM-1		Polyclonal Goat IgG	1:100	R&D Systems	AF3628
Anti-CD68		FA-11	1:200	Abcam	ab53444
Anti-LYVE1		EPR21771	1:200	Abcam	ab218535
Goat anti-rat IgG (H+L)	Alexa Fluor™ 647		1:200	Invitrogen	A-21247
Goat anti-rabbit IgG (H+L)	Alexa Fluor™ 568		1:200	Invitrogen	A-11011
Donkey anti-goat IgG (H+L)	Alexa Fluor™ 488		1:200	Invitrogen	A-11055
Donkey anti-rabbit IgG (H+L)	Alexa Fluor™ 568		1:200	Invitrogen	A10042
Donkey Anti-Rat IgG H&L	Alexa Fluor® 647		1:200	Abcam	ab150155

Supplementary Table 1. List of used antibodies.
