# Supplementary material for: DPPIV+ fibro-adipogenic progenitors form the niche of adult skeletal muscle self-renewing resident macrophages
Source: Nat Commun. 2023 Dec 13;14:8273. doi: 10.1038/s41467-023-43579-3 (PMC10719395; doi:10.1038/s41467-023-43579-3)
Supplement: Supplementary file 3 — Reporting Summary [file 41467_2023_43579_MOESM3_ESM.pdf]

Corresponding author(s): Fabio Rossi, Farshad Babaeijandaghi

Last updated by author(s): Aug 28, 2023

Reporting Summary

Nature Portfolio wishes to improve the reproducibility of the work that we publish. This form provides structure for consistency and transparency in reporting. For further information on Nature Portfolio policies, see our Editorial Policies and the Editorial Policy Checklist.

Statistics

For all statistical analyses, confirm that the following items are present in the figure legend, table legend, main text, or Methods section.

n/a	Confirmed	
☐	☒	The exact sample size (*n*) for each experimental group/condition, given as a discrete number and unit of measurement
☐	☒	A statement on whether measurements were taken from distinct samples or whether the same sample was measured repeatedly
☐	☒	The statistical test(s) used AND whether they are one- or two-sided<br>*Only common tests should be described solely by name; describe more complex techniques in the Methods section.*
☐	☒	A description of all covariates tested
☐	☒	A description of any assumptions or corrections, such as tests of normality and adjustment for multiple comparisons
☐	☒	A full description of the statistical parameters including central tendency (e.g. means) or other basic estimates (e.g. regression coefficient) AND variation (e.g. standard deviation) or associated estimates of uncertainty (e.g. confidence intervals)
☐	☒	For null hypothesis testing, the test statistic (e.g. *F*, *t*, *r*) with confidence intervals, effect sizes, degrees of freedom and *P* value noted<br>*Give P values as exact values whenever suitable.*
☒	☐	For Bayesian analysis, information on the choice of priors and Markov chain Monte Carlo settings
☒	☐	For hierarchical and complex designs, identification of the appropriate level for tests and full reporting of outcomes
☒	☐	Estimates of effect sizes (e.g. Cohen's *d*, Pearson's *r*), indicating how they were calculated

Our web collection on statistics for biologists contains articles on many of the points above.

Software and code

Policy information about availability of computer code

Data collection	Magelan pro v7.3, Quantasoft v1.7.4.0917, NIS Elements v5.11.03, CytExpert v2.4, ZEN Blue v3.4.91.00000, BD FACS™ Software v1.0.0.650, BD FACSDiva™ V6.1.3
Data analysis	bcl2fastq v2.20, RNA-Seq Alignment v1.1.1, R v4.1.2, Seurat v4.3.0, R v3.6.2, GraphPad Prism v9, Adobe Illustrator v27.7, Adobe Photoshop v24.6.0, Adobe Acrobat Pro v3.6.20320.0, ImageJ v2.0.0-rc-69/1.52p, FlowJo Engine v5.00000, SCTransform v2

For manuscripts utilizing custom algorithms or software that are central to the research but not yet described in published literature, software must be made available to editors and reviewers. We strongly encourage code deposition in a community repository (e.g. GitHub). See the Nature Portfolio guidelines for submitting code & software for further information.

Data

Policy information about availability of data

All manuscripts must include a data availability statement. This statement should provide the following information, where applicable:

- Accession codes, unique identifiers, or web links for publicly available datasets
- A description of any restrictions on data availability
- For clinical datasets or third party data, please ensure that the statement adheres to our policy

All raw RNAseq data are deposited in the Gene Expression Omnibus (GEO) database under the accession ID GSE241832.

Research involving human participants, their data, or biological material

Policy information about studies with human participants or human data. See also policy information about sex, gender (identity/presentation), and sexual orientation and race, ethnicity and racism.

Reporting on sex and gender	This study did not involve human research participants.
Reporting on race, ethnicity, or other socially relevant groupings	This study did not involve human research participants.
Population characteristics	This study did not involve human research participants.
Recruitment	This study did not involve human research participants.
Ethics oversight	This study did not involve human research participants.

Note that full information on the approval of the study protocol must also be provided in the manuscript.

Field-specific reporting

Please select the one below that is the best fit for your research. If you are not sure, read the appropriate sections before making your selection.

☒ Life sciences ☐ Behavioural & social sciences ☐ Ecological, evolutionary & environmental sciences

For a reference copy of the document with all sections, see nature.com/documents/nr-reporting-summary-flat.pdf

Life sciences study design

All studies must disclose on these points even when the disclosure is negative.

Sample size	Sample size calculation was not carried out for this study. Instead, our lab's extensive experience with similar experiments guided the selection of sample sizes, taking into account the expected and observed data variability within each group as well as the differences between groups.
Data exclusions	Data exclusions were made for scRNAseq experiments based on: 1) doublet detection through cell hashing (using 0.99 quantile as a threshold for positive hashing, if applicable); 2) quality control metrics for each cell. A cell is removed if it expresses less than 200 genes, retains extreme number of UM Is compare to other cells in the dataset, and more than 10-15% of its transcripts are mitochondrial transcripts. No other data were excluded from analysis.
Replication	The figure legends specify the number of independent experiments conducted. Data from various experimental replicates were combined for presentation and analysis. No data were omitted from the study unless explicitly stated in the manuscript.
Randomization	We used age- and sex-matched littermates randomly assigned to different experimental groups wherever possible.
Blinding	The investigators were not able to conduct blinding during the experiments due to the same investigator performing flow cytometry, ELISA, or ddPCR. However, for the analysis of histological samples, a different investigator handled quantification, and blinding procedures were implemented whenever feasible.

Reporting for specific materials, systems and methods

We require information from authors about some types of materials, experimental systems and methods used in many studies. Here, indicate whether each material, system or method listed is relevant to your study. If you are not sure if a list item applies to your research, read the appropriate section before selecting a response.

Materials & experimental systems

n/a	Involved in the study
☐	☒ Antibodies
☒	☐ Eukaryotic cell lines
☒	☐ Palaeontology and archaeology
☐	☒ Animals and other organisms
☒	☐ Clinical data
☒	☐ Dual use research of concern
☒	☐ Plants

Methods

n/a	Involved in the study
☒	☐ ChIP-seq
☐	☒ Flow cytometry
☒	☐ MRI-based neuroimaging

Antibodies

Antibodies used

The full list of antibodies is provided in Table 1.

Validation

Anti-TIM4 (BioLegend, # 130010): This monoclonal antibody recognizes Mouse TIM4-Ig fusion protein. It has been tested by flow cytometric analysis of Balb/c peritoneal macrophages. Rat IgG2a, κ PE/Cyanine7 was used as an isotype control (https://www.biolegend.com/fr-ch/products/pe-cyanine7-anti-mouse-tim-4-antibody-11944)

Anti-LYVE1 (Invitrogen, # 48-0443-82): This monoclonal antibody has been tested by flow cytometric analysis of BAF3 cell line transfected with LYVE-1 (https://www.thermofisher.com/antibody/product/LYVE1-Antibody-clone-ALY7-Monoclonal/48-0443-82)

Anti-CD45 (BioLegend, # 103128): This monoclonal antibody has been tested by flow cytometric analysis of C57BL/6 mouse splenocytes (https://www.biolegend.com/fr-ch/products/alexa-fluor-700-anti-mouse-cd45-antibody-3407)

Anti-CD11b (BioLegend, # 101257): This monoclonal antibody has been tested by flow cytometric analysis of C57BL/6 mouse bone marrow cells (https://www.biolegend.com/fr-ch/products/brilliant-violet-605-anti-mouse-human-cd11b-antibody-7637)

Anti-CD170 (Siglec-F) (Invitrogen, # 46-1702-82): This monoclonal antibody has been tested by flow cytometric analysis of mouse thioglycolate-elicited peritoneal exudate cells (https://www.thermofisher.com/antibody/product/CD170-Siglec-F-Antibody-clone-1RNM44N-Monoclonal/46-1702-82)

Anti-CD170 (Siglec-F) (BD Pharmingen™, # 562680): This monoclonal antibody has been tested by multicolor flow cytometric analysis of Siglec-F expression on BALB/c mouse bone marrow cells. Alexa Fluor® 647 Rat IgG2a, κ Isotype Control (Cat. No. 557690; Left Panel) was used as a control (https://www.bdbiosciences.com/en-us/products/reagents/flow-cytometry-reagents/research-reagents/single-color-antibodies-ruo/alexa-fluor-647-rat-anti-mouse-siglec-f.562680)

Anti-MHC Class II (I-A/I-E) (eBioscience™, # 48-5321-82): This monoclonal antibody has been tested by flow cytometric analysis of mouse splenocytes (https://www.thermofisher.com/antibody/product/MHC-Class-II-I-A-I-E-Antibody-clone-M5-114-15-2-Monoclonal/48-5321-82)

Anti-CD192 (CCR2) (BioLegend, # 150627): This monoclonal antibody has been tested by flow cytometric analysis of C57BL/6 bone marrow. Rat IgG2b, κ APC was used as an isotype control (https://www.biolegend.com/fr-ch/products/apc-anti-mouse-cd192-ccr2-antibody-17676)

Anti-LY6G (BD Pharmingen™, # 560602): This monoclonal antibody has been tested by flow cytometric analysis of bone marrow cells from C57BL/6 mice. Rat IgG2a, κ was used as an isotype control (https://www.bdbiosciences.com/en-us/products/reagents/flow-cytometry-reagents/research-reagents/single-color-antibodies-ruo/percp-cy-5-5-rat-anti-mouse-ly-6g.560602)

Anti-F4/80 (nvitrogen, # 12-4801-82): This monoclonal antibody has been tested by flow cytometric analysis of mouse resident peritoneal exudate cells (https://www.thermofisher.com/antibody/product/F4-80-Antibody-clone-BM8-Monoclonal/12-4801-82)

Anti-Sca-1 (eBioscience™, # 25-5981-82 and 11-5981-82): This monoclonal antibody has been tested by flow cytometric analysis of mouse splenocytes (https://www.thermofisher.com/antibody/product/Ly-6A-E-Sca-1-Antibody-clone-D7-Monoclonal/25-5981-82).

Anti-CD31 (BD Pharmingen™, # 551262): This monoclonal antibody has been tested by multicolor flow cytometric analysis of CD31 expression on mouse bone marrow cells. APC Rat IgG2a, κ was used as an Isotype Control (https://www.bdbiosciences.com/en-us/products/reagents/flow-cytometry-reagents/research-reagents/single-color-antibodies-ruo/apc-rat-anti-mouse-cd31.551262)

Anti-DPP4 (BioLegend, # 137810): This monoclonal antibody has been tested by flow cytometric analysis of C57BL/6 splenocytes. Rat IgG2a, κ PE/Cyanine7 was used as an Isotype Control (https://www.biolegend.com/fr-ch/products/pe-cyanine7-anti-mouse-cd26-dpp-4-antibody-10368)

Anti-CD45.2 (eBioscience™, # 17-0454-82): This monoclonal antibody has been tested by flow cytometric analysis of SJL and BALB/c splenocytes. Mouse IgG2a K was used as an Isotype Control (https://www.thermofisher.com/antibody/product/CD45-2-Antibody-clone-104-Monoclonal/17-0454-82)

Anti-CD45.1 (eBioscience™, # 11-0453-85): This monoclonal antibody has been tested by flow cytometric analysis of SJL and BALB/c splenocytes. Mouse IgG2a K was used as an Isotype Control (https://www.thermofisher.com/antibody/product/CD45-1-Antibody-clone-A20-Monoclonal/11-0453-85)

I

Streptavidin (BioLegend, # 405235): Streptavidin has been tested by flow cytometric analysis of human peripheral blood lymphocytes stained with biotinylated CD3. Biotinylated mouse IgG1 was used as an isotype control (https://www.biolegend.com/fr-ch/products/alexa-fluor-488-streptavidin-9304)

Streptavidin (eBioscience™, # 48-4317-82): This has been tested by flow cytometric analysis of mouse splenocyte cells stained Anti-Human/Mouse CD45R (B220) Biotin. Mouse IgG2b kappa was used as an Isotype Control (https://www.thermofisher.com/order/catalog/product/48-4317-82)

Anti- DPP4 (R&D Systems, # AF954): This polyclonal antibody has been tested on immersion fixed frozen sections of mouse thymus. Specific staining was localized to lymphocytes (https://www.rndsystems.com/products/mouse-dppiv-cd26-antibody_af954)

Anti-CD31/PECAM-1 (R&D Systems, # AF3628): This polyclonal antibody has been tested on immersion fixed frozen sections of mouse embryo (E13.5). Specific staining was localized to developing endothelium (https://www.rndsystems.com/products/human-mouse-rat-cd31-pecam-1-antibody_af3628)

Anti-CD68 (Abcam, # ab53444): This monoclonal antibody has been tested on RAW 264.7 cells (https://www.abcam.com/products/primary-antibodies/cd68-antibody-fa-11-ab53444.html?productWallTab=ShowAll)
Anti-LYVE1 (Abcam, # ab218535): This antibody has been tested on frozen mouse stomach tissue showing positive staining of the endothelium of lymph vessels (https://www.abcam.com/products/primary-antibodies/lyve1-antibody-epr21771-ab218535.html#lb)

Goat anti-rat IgG (H+L) (Invitrogen, A-21247): Immunofluorescence analysis of this antibody was performed using A549 cells stained with alpha Tubulin (YL1/2) Rat Monoclonal Antibody (https://www.thermofisher.com/antibody/product/Goat-anti-Rat-IgG-H-L-Cross-Adsorbed-Secondary-Antibody-Polyclonal/A-21247)

Goat anti-rabbit IgG (H+L) (Invitrogen, A-11011): Immunofluorescence analysis of this antibody was performed using HeLa cells stained with alpha Tubulin Rabbit Polyclonal Antibody (https://www.thermofisher.com/antibody/product/Goat-anti-Rabbit-IgG-H-L-Cross-Adsorbed-Secondary-Antibody-Polyclonal/A-11011)

Donkey anti-goat IgG (H+L) (Invitrogen, # A-11055): This antibody has been tested by flowcytometric analysis of OXTR expression on Ishikawa cells (human endometrial adenocarcinoma cell line) (https://www.thermofisher.com/antibody/product/Donkey-anti-Goat-IgG-H-L-Cross-Adsorbed-Secondary-Antibody-Polyclonal/A-11055)

Donkey anti-rabbit IgG (H+L) (Invitrogen, # A10042): Immunofluorescence analysis of this antibody was performed using HepG2 cells stained with alpha-1 antitrypsin Rabbit Polyclonal Primary Antibody (https://www.thermofisher.com/antibody/product/Donkey-anti-Rabbit-IgG-H-L-Highly-Cross-Adsorbed-Secondary-Antibody-Polyclonal/A10042)

Donkey Anti-Rat IgG H&L (Abcam, # ab150155): Immunofluorescence analysis of this antibody was performed using HeLa cells stained with Anti-Tubulin antibody Primary Antibody (https://www.abcam.com/products/secondary-antibodies/donkey-rat-igg-hl-alexa-fluor-647-preadsorbed-ab150155.html#lb)

Anti-CD11c (AbLab, # 48-0051-01 and #67-0051-01): This monoclonal antibody has been tested on C57BL/6 mouse splenocytes.

Anti-CD106 (AbLab, # 31-0057-01): This monoclonal antibody has been tested on C57BL/6 mouse Bone Marrow.

Anti-Fcgamma Receptor (AbLab, # 21-0041-05): Purified antibody has been tested on isolated C57BL/6 mouse splenocytes. Staining of the antibody was detected with anti-rat IgG.

Animals and other research organisms

Policy information about studies involving animals; ARRIVE guidelines recommended for reporting animal research, and Sex and Gender in Research

Laboratory animals	Animals were housed in in a specific pathogen-free facility with a temperature range of 21-23°C, humidity levels between 40-60%, and a standard 12-hour light/12-hour dark cycle. Adult mice of both sexes, aged between 7 and 24 weeks were used in this study. We used age- and sex-matched littermates randomly assigned to different experimental groups wherever possible. C57BL/6J (#000664), B6 Cd45.1 (#002014), ROSA-DTA (#009669), and PDGFRαEGF (#007669) mice were purchased from The Jackson Laboratory. C57BL/6 mice expressing GFP ubiquitously from a cytomegalovirus–β-actin hybrid promoter were a gift of I. L. Weissman (Stanford University). To generate Csf1null/flox (Csf1flox) mice, non-tissue-specific null alleles of Csf1 were obtained through a cross between Tie2-Cre (The Jackson Laboratory, #008863) with Csf1 flox/flox mice (a gift of S.A. Werner and J.X. Jiang, University of Texas Health Science Center at San Antoni). Tie2-Cre recombinase activity is detected in endothelial tissues, as well as reproductive tissues, leading to the germline deletion of the floxed allele. Subsequently, these Csf1null/flox (Csf1flox) mice were crossed with PDGFR-Cre-ERT2 mice (The Jackson Laboratory, #032770) 56 to conditionally delete the Csf1 floxed allele in FAPs.
Wild animals	No wild type animals were used in this study.
Reporting on sex	Both sexes were used in this study. We used age- and sex-matched littermates randomly assigned to different experimental groups wherever possible.
Field-collected samples	No field collected samples were used in this study.

Ethics oversight	All animal procedures were carried out in accordance with the University of British Columbia Animal Care Committee guidelines under experimental protocol numbers A18-0314, A22-0245, and A19-0316.

Note that full information on the approval of the study protocol must also be provided in the manuscript.

Flow Cytometry

Plots

Confirm that:

☒ The axis labels state the marker and fluorochrome used (e.g. CD4-FITC).

☒ The axis scales are clearly visible. Include numbers along axes only for bottom left plot of group (a 'group' is an analysis of identical markers).

☒ All plots are contour plots with outliers or pseudocolor plots.

☒ A numerical value for number of cells or percentage (with statistics) is provided.

Methodology

Sample preparation	Mice were initially anesthetized via intraperitoneal injection of 2,2,2-tribromoethyl alcohol (Aldrich T4840-2). To avoid confounding results, prior to tissue collection, mice were perfused with 15 mL of warm PBS to remove circulating cells from blood vessels. Following removing the fascia, skeletal muscle from both hindlimbs of the mice were then carefully dissected. A mixture of Collagenase D (Roche; 1.5 U ml-1; # 11088882001) and Dispase II (Roche; 2.4 U ml-1; # 04942078001), and 10mM $CaCl_2$ was added to the samples and was incubated at 37°C for 60 minutes. During the incubation period, the muscle mixtures were vortexed initially and then once every 20 minutes, for a total of four times. Preparations were passed through a 40µm cell strainer (Falcon™, # 352340) and washed with PBS. This filtration created a single suspension of cells which was then centrifuged for 7 minutes at 550g. In preparation for flow cytometry, blocking buffer, composed of 10% goat serum, flow cytometry staining (FACS) buffer (PBS, 2% FBS, 0.4% EDTA) and 1:200 Anti-Fcγ Receptor (AbLab, clone 24G2, # 21-0041-05) were added to each falcon tube containing the single cell suspensions. A cocktail of antibodies (Table 1) was then added to each cell suspension in the appropriate dilutions.
Instrument	Stained cells were washed and analyzed using a LSRII (Becton Dickinson) or CytoFLEX (Beckman Coulter). Cell sorting was performed using a FACSAri or Influx cell sorter (Becton Dickinson).
Software	BD FACSDiva™ Software or CytExpert
Cell population abundance	The percentage of each population is provided in the figures.
Gating strategy	The gating strategy is shown in each figure.

☒ Tick this box to confirm that a figure exemplifying the gating strategy is provided in the Supplementary Information.
